# Water Soluble Cyclophosphamide Adducts of Rhodium(II) Keto-Gluconate and Glucuronate. Synthesis, Characterization and *In Vitro* Cytostatic Assays

**DOI:** 10.1155/MBD.1999.19

**Published:** 1999

**Authors:** Eric de Souza Gil, Maria Inês de Almeida Gonçalves, Elizabeth Igne Ferreira, Szulin Ber Zyngier, Renato Najjar

**Affiliations:** 1 Faculty of Pharmaceutical Sciences University of São Paulo SP 05389-970 Brazil; 2 Institute of Biomedical Sciences Department of Pharmacology University of São Paulo SP Brazil; 3 Institute of Chemistry University of São Paulo SP Brazil

## Abstract

The synthesis, characterization and biological assays of two new rhodium carboxylate sugar derivatives and respective cyclosphosphamide adducts are described. The compounds, characterized by ^13^C and ^1^H NMR, infrared and UV-visible spectra, presented high water solubility and hydration grades were confirmed given the concordance between thermal and CHN analyses. The adducts were active *in vitro* against K-562 cells.


### WATER SOLUBLE CYCLOPHOSPHAMIDE ADDUCTS OF RHODIUM(II) KETO-GLUCONATE AND GLUCURONATE. SYNTHESIS, CHARACTERIZATION AND *IN VITRO* CYTOSTATIC ASSAYS

Eric de Souza Gil*[1], Maria Inês de Almeida Gonçalves[1],

Elizabeth Igne Ferreira[1], Szulin Ber Zyngier[2] and Renato Najjar[3]

[1] Faculty of Pharmaceutical Sciences, University of São Paulo, SP-Brazil 05389-970
[2] Institute of Biomedical Sciences, Department of Pharmacology, University of São Paulo, SP-Brazil
[3] Institute of Chemistry, University of São Paulo, SP-Brazil

### ABSTRACT
The synthesis, characterization and biological assays of two new rhodium carboxylate sugar derivatives and respective cyclosphosphamide adducts are described. The compounds, characterized by $^{13}C$ and $^{1}H$ NMR, infrared and UV-visible spectra, presented high water solubility and hydration grades were confirmed given the concordance between thermal and CHN analyses. The adducts were active *in vitro* against K-562 cells.

### INTRODUCTION
Ever since Rosenberg *et al.*[1] introduced cisplatin in tumour disease therapeutics, researchers became increasingly interested in this field, giving rise to a number of published findings on platinum group metal complexes. In 1972, Bear and co-workers reported that rhodium (II) carboxylates present anti-tumoural activity[2]. Albeit the promising start, interest in this class of compounds as anti-tumoural agents has somewhat decreased mostly in view of significant toxicity levels. Currently, a number of papers have been issued in an attempt to identify less toxic derivatives [3-8].

One of the best means of obtaining chemotherapeutical metal complexes is to synthesize adducts using ligand molecules that are, by their very nature, biologically active. To this effect, cyclophosphamide (CP) *(Fig.1)* has been used to obtain adducts with rhodium(II) carboxylates. The compounds were submitted to biological assays and results indicated that the complexes were not active [9]. This might be partially due to the fact that these present somewhat low water solubility levels. With views to obtaining compounds that present the appropriate partition coefficient for biological assays, two new rhodium(II) derivatives, $Rh_2(GU)_4$ and $Rh_2(KG)_4$, rhodium glucuronate and rhodium keto-gluconate *(Fig.1)* respectively, plus their adducts with CP, have been synthesized and characterized.

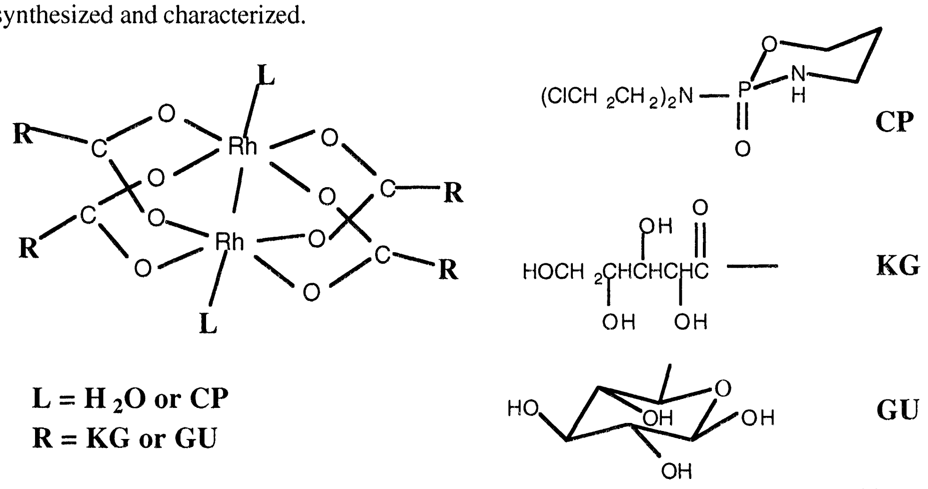

Fig. 1 - Structure of the new rhodium(II) carboxylates and cyclophosphamide adducts

### MATERIALS AND METHODS
*Chemicals*

Rhodium chloride hydrate, hemicalcium D-gluconate and hemicalcium 2-keto-D-gluconate dihydrate were purchased from Aldrich, sodium D-glucuronate and Sephadex C-25 from Sigma, whilst Sephadex G-25 was acquired from Pharmacia.

*Instruments*
$^1H$-NMR, $^{13}C$-NMR and $^{31}P$-NMR analyses were carried out in an Bruker Advanced 300 MHz spectrometer, utilizing $D_2O$ as solvent. IR spectra were recorded on a 1750 FTIR-Perkin-Elmer analyzer, model 783, employing KBr pellets. Elemental analyses were performed on 240 B or 2400 Perkin-Elmer analyzers. UV-visible spectra were recorded on a Hitachi U-3000 spectrometer whilst for thermal analyses purposes, a Shimadzu TGA-50 was utilized.
*Synthesis*
Tetrakis-dirhodium (II) glucuronate and tetrakis-dirhodium (II) keto-gluconate, two functional isomers whose molecular formula is $(Rh_2(C_5H_9O_5COO)_4($ were prepared mixing 2.1 *N* solutions of the respective sugar salts in water, with an 1 mol.L$^{-1}$ ethanolic solution of $RhCl_3.xH_2O$, under reflux at 70°C during 4-6 hours. The resultant green solution was submitted to column chromatography with G-25 and C-25 Sephadex, rendering a blue, limpid solution. The products presented some sugar properties, such as solubility and thermostability. The derivatives were subsequently freeze-dried and resultant respective yields were 25 and 33%. Adducts were prepared mixing 1 mol.L-1 solutions of free rhodium carboxylates with a 3 mol.L-1 water methanol (1:1) solution of cyclophosphamide. The green solution obtained was likewise submitted to freeze drying, the powder washed with cold dichloromethane and dried, under vacuum. The yields of both adducts were approximately 80%.
*Biological tests*
*Cytotoxicity*
K562 human leukaemia cells were cultured in RPMI media supplemented by 10% fetal serum. To test drug effectiveness, $10^5$ cells were sown onto a 24 well plate containing 1 mL of the same medium. The samples were added to the wells so as to obtain 132 μg/L and 266 μg/L final concentrations. A sodium bicarbonate solution was added to the control wells. Twenty-four hours after adding the compound, well contents were collected, centrifuged, stained with Trypan Blue and cells were counted.
Resultant data were compared by means of the statistic chi square test[10]. When $P \leq 0.05$, differences were deemed to be statistically significant.
*Preliminar toxicity assay*
Toxicity was investigated in nine different groups of eight healthy male Balb-C mice, with a single *ip* dose of up to 200mg/kg. Death and/or toxic effects were sought for, during 60 days.

### RESULTS AND DISCUSSION

Table 1 presents the results encountered in carbon, nitrogen and hydrogen analyses. Percentages were compatible with the compounds' high water affinity.
The first inflection of TGA curves *(Fig.2)* is relative to water lost to $Rh_2(KG)_4xH_2O$ and the hydration grade ($x=6\text{-}8\ H_2O$) depends on freeze drying conditions.

TABLE 1 - CHN analyses

| | Experimental (%) | | | Calculated (%) | | |
|---|---|---|---|---|---|---|
| COMPOUND | C % | H% | N % | C% | H% | N % |
| $Rh_2(GU)_4\ 6H_2O$ | 26.62 | 4.62 | - | 26.53 | 4.45 | - |
| $Rh_2(KG)_4\ 8H_2O$ | 25.51 | 4.63 | - | 25.68 | 4.67 | - |
| $Rh_2(GU)_4(CP)_2$ | 27.55 | 4.89 | 3.35 | 27.75 | 5.03 | 3.41 |
| $Rh_2(KG)_4(CP)_2$ | 28.35 | 4.79 | 3.15 | 28.37 | 4.89 | 3.48 |

Sugar salt, rhodium compound and respective CP adduct's major infrared assignments are depicted in Table 2. Coordination modes are forecasted as of the stretched, asymmetric (~1600 cm$^{-1}$) and symmetric (~1400 cm$^{-1}$) values, of the coordinated carboxylate groups. Average Δ(asym-sym) values under 200 cm$^{-1}$ allow for the discarding of the hypothesis of occurrence of complex's monodentate coordination [11]. Within the 3500-3000 cm$^{-1}$ range, the free D-glucuronic acid presents three broad bands centered at 3400, 3280 and 3160 cm$^{-1}$ due to OH stretching. The first two are attributed to intermolecular interactions and the latter might be related to an intramolecular hydrogen bond [12]. Usually these bands are shifted to a higher frequency when the sugar ligands coordinate to metals. It is worth noting that such shifts may be influenced by the metal charge and consequently by the covalent character of the metal-ligand bond. For instance, the $Rh_2(GU)_4$ highest energy band at 3400 cm$^{-1}$ in sugar free acid, shifts to 3600 cm$^{-1}$ in its sodium salt and to 3470 cm$^{-1}$ in calcium salt [12], remaining at approximately 3400 cm$^{-1}$ for the rhodium (II) dimer. This suggests that the intermolecular hydrogen bonds of the free acids are analogous to those observed in the rhodium derivatives.

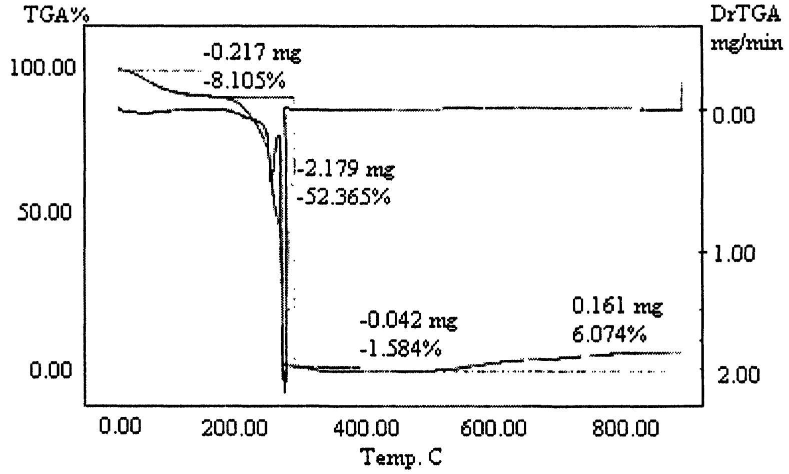

Fig.2 - TGA Curves of $Rh_2(KG)_4.xH_2O$

TABLE 2 - IR assignments

| Na(GU) | Ca(GU)$_2$ | Rh$_2$(GU)$_4$ | Rh$_2$(KG)$_4$ | Rh$_2$(GU)$_4$(CP)$_2$ | Rh$_2$(KG)$_4$(CP)$_2$ | Assignments |
|---|---|---|---|---|---|---|
| 3602 | 3470 | 3412 | 3394 | 3424 | 3395 | $\nu_{O-H}$ |
| - | - | 1605 | 1613 | 1606 | 1614 | $\nu_{OCO^-}$ asym |
| - | - | 1433 | 1424 | 1433 | 1425 | $\nu_{OCO^-}$ sym |
| - | - | **172** | **189** | **173** | **189** | ($\nu_{asym}$-$\nu_{sym}$) |

TABLE 3 - Rhodium carboxylates sugar derivative $^1H$ and $^{13}C$ NMR shifts.

| | |
|---|---|
|  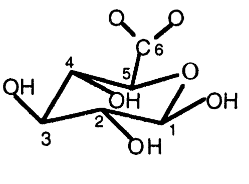 | $Rh_2(GU)_4$ |
| $^1$H-RMN ($D_2O$, $\delta$) | 4.95 (d, H1), 4.38 (d, H1'), 3.88 (d, H5), 3.43, 3.39 (H3, H5'), 3.31-2.96 (H2, H4, H3', H4', H2') |
| $^{13}$C-RMN ($D_2O$, ppm) | 189.5 (C6), 188.5 (C6'), 95.94 (C1), 91.96 (C1'), 74.72 (C3'), 74.61 (C5'), 73.76 (C2'), 73.65 (C3), 71.85 (C2), 71.33 (C5), 70.71 (C4), 70.58 (C4') |
|  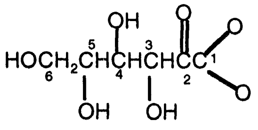 | $Rh_2(KG)_4$ |
| $^1$H-RMN ($D_2O$, $\delta$) | 3.75 (H3), 3.70 (d, H6), 3.57-3.50 (m, H5), 3.52 (H4), 3.45-3.40 (d, H6). |
| $^{13}$C-RMN ($D_2O$, ppm) | 56 (C1), 97.30 (C2), 95.9 (C3), 81 (C4), 78.06 (C4'), 73.30 (C5), 69.9 (C5'), 68.80 (C6), 63.95 (C6') |

Additional splitting is observed in the $^1$H-NMR spectra of the rhodium compounds as compared to other derivatives such as the sodium and calcium salts of KG and GU. The extensive number of conformations in sugar complexes supports this finding. The $^1$H and $^{13}$C chemical shifts (Table 3) of these sugar compounds when compared with their salt derivatives[14], present different characteristics, specially those related to the decrease in the ionic character of the M-O bond.

TABLE 4 - $^{1}H$, $^{13}C$ and $^{31}P$ NMR shifts of GUCP

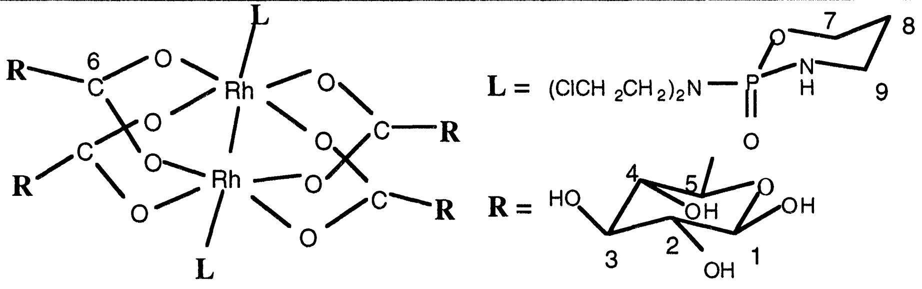

| | |
|---|---|
| $^{1}H$ NMR ($D_2O$, δ) | 4.95(d,H1), 4.38-3.07(H5,H1',H7,H10,H11,H3,H3',H2,H4,H4',H5',H2, H2'), 1.89-1.84(H8) |
| $^{13}C$ NMR ($D_2O$, ppm) | 189.5(C6), 188.5(C6'), 95.95(C1), 95.61(C1'), 74.73(C3'), 74.60(C5'), 73.44(C2'), 73.24(C3), 71.85(C2), 71.29(C5), 70.73(C4), 70.61(C4'), 62.54(C7), 62.46(C7'), 49.38(C10), 49.05(C10'), 42.39(C11), 42.81(C11'), 38.90(C9), 26.22(C8), 26.13(C8') |
| $^{31}P$ NMR ($D_2O$, ppm) | 13.57, -1.34, -12.70 |

Such diverse characteristics are better demonstrated by means of $^{13}C$ spectral analyses whereby carboxyl carbon signals shift to the 13 ppm downfield in the rhodium (II) complexes. This might be due to the fact that as the carboxylic oxygen coordinates to rhodium(II), electronic displacements occur, deshielding the carbon atom. Therefore this is in agreement with the infrared results, suggesting a higher covalent character to the Rh-O bond.

TABLE 5 - $^{1}H$, $^{13}C$ and $^{31}P$ NMR shifts of KGCP

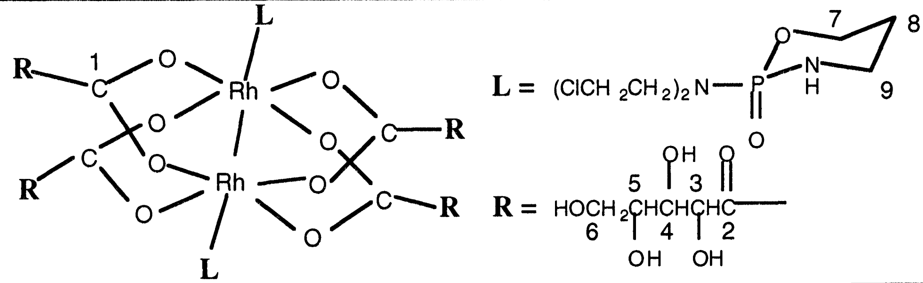

| | |
|---|---|
| $^{1}H$ NMR ($D_2O$, δ) | 4.95(d,H1), 4.38-3.07(H5,H1',H7,H10,H11,H3,H3',H2,H4,H4',H5',H2, H2'), 1.89-1.84(H8) |
| $^{13}C$ NMR ($D_2O$, ppm) | 189.5(C1), 188.5(C1'), 95.95(C2), 95.61(C2'), 74.73(C3'), 74.60(C5'), 73.44(C2'), 73.24(C3), 71.85(C2), 71.29(C5), 70.73(C4), 70.61(C4'), 62.54, 62.46(C7,C7'), 49.38, 49.05(C10, C10'), 42.39, 42.81(C11,C11'), 38.90(C9), 26.22, 26.13(C8,C8') |
| $^{31}P$ NMR ($D_2O$, ppm) | 1355, 9.35, 0.41, -1.35, -1.63, -12.70 |

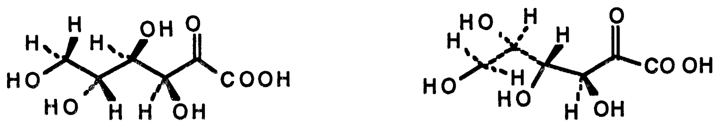

flat zig-zag

Fig. 3- Possible conformations of KG

All signals in the cyclic derivative (GU) related to both anomeric forms α and β (the latter represented by ') were identified and carefully assigned as per literature data [9,14-18]. Two possible conformations [15-17] (Fig.3) explained the additional shifts (represented by ') encountered in keto-gluconate compounds. This effect was most intense in carbon 6.

In addition, with reference to the keto-gluconate compounds, a carbonyl group that presented an atypical signal at approximately *c.a.* 97 ppm was identified. In conclusion, the displacement was probably due to a keto-enolic equilibrium. Nevertheless, it is worth emphasizing that other than $^{31}P$ NMR, few important shifts were observed after axial CP coordination in the $^{1}H$ and $^{13}C$ NMR spectra.

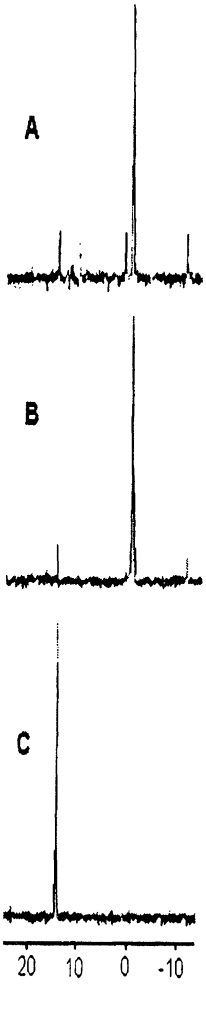

Fig. 4 - $^{31}P$ spectra of free CP (C) and their rhodium adducts:[$Rh_2(KG)_4(CP)_2$] (A) and [$Rh_2(GU)_4(CP)_2$] (B)

$^{1}H$, $^{13}C$ and $^{31}P$ NMR shift data are pictured in Table 4, while Table 5 presents those for $Rh_2(RCOO)_4(CP)_2$, R=GU and KG, respectively.

Figure 4 depicts the $^{31}P$ NMR spectra for both adducts and the free CP.

Additional peaks in the spectra of adducts were observed in $^{31}P$ NMR. The first signal at +13.55 ppm is assigned to free CP ligand and agrees with the dissociation of the adducts. A downfield shift of the $^{31}P$ peaks for the ($Rh_2(KG)_4(CP)_2$( and ($Rh_2(GU)_4(CP)_2$( was expected, but not confirmed [9]. The signal of these complexes appeared at −1.35 ppm and 1.34 ppm. This might be due to a prompt dissociation of the adduct, forming $Rh_2(RCOO)_4$ and CP in water solution in view of the higher donor character of $D_2O$ in relation to that of $CDCl_3$. The third peak, at −12.7 ppm, is probably related to ($Rh_2(RCOO)_4(CP)_2(H_2O)$(upon partial dissociation.

Electronic spectra (Table 6) exhibit the four characteristic peaks, "the finger print" of rhodium carboxylates.

Existing literature [9] reports that cyclophosphamide is not a ligand that presents high affinity with rhodium(II) carboxylates. A link *via* the oxygen P=O was involved when adducts were isolated. In addition, should this have been due to a nitrogen link, the compound would most probably have been pink in colour, although there are exceptions. In support of this hypothesis, the 590 nm band in UV/vis spectra (Table 6) suggests, as observed in other ligands, a coordination through oxygen. Although data indicate that oxygen coordination is most probable, the alternative nitrogen coordination *via* hydrogen bonds could not be discarded.

### *BIOLOGICAL TESTS*

Table 7 presents the results of cytotoxic activity. Data was submitted to the statistical chi squared $\chi^2$ test.

Although all compounds presented higher *in vitro* activity in relation to the control, no deaths or toxic effects were observed along the 60 days of *in vivo* tests using doses of up to 200mg/kg. The group treated with adducts showed a significant increase of cytotoxicity *in vitro*, mostly in the highest doses. The most promising in the series, against the cell line tumour used, was the KG derivative. On the other hand, it is worth noting that $Rh_2(KG)_4(CP)_2$ presented higher activity than the CP and $Rh_2(KG)_4$ from which it is derived. Possibly, once these adducts are administered, a gradual dissociation occurs before acting upon the cancer cells. Should this be the case, both rhodium carboxylates and CP meet each other's requirements as carriers, promoting a synergistic effect *in vivo*. The relevance of this effect requires further investigation and will be subject to forthcoming studies.

TABLE 6 - UV-visible assignments

| peak | $Rh_2(RCOO)_4$ | $Rh_2(RCOO)_4(CP)_2$ |
|---|---|---|
| 1 | ~ 590 nm | 590 nm |
| 2 | ~ 460 nm | 438 nm |
| 3 (shoulder) | ~ 240 nm | 225 nm |
| 4 | ~ 200 nm | 200 nm |

R= KG ou GU

TABLE 7 - Cytotoxic activity

| Compound | Dead Cells (x $10^4$ cells/mL) | | Living Cells (x $10^4$ cells/mL) | | Total (x $10^4$ cells/mL) | | Dead Cells (%) | |
|---|---|---|---|---|---|---|---|---|
| | 132 μg/L | 266 μg/L | 132 μg/L | 266μg/L | 132 μg/L | 266 μg/L | 132 μg/L | 266 μg/L |
| $Rh_2(GU)_4$ | 0 | 1 | 5 | 3 | 5 | 4 | 0 | 25 |
| $Rh_2(KG)_4$ | 0 | 1 | 6 | 5 | 6 | 6 | 0 | 17 |
| $Rh_2(GU)_4(CP)_2$ | 1 | 3 | 4 | 2 | 5 | 5 | 20 | 60 |
| $Rh_2(KG)_4(CP)_2$ | 4 | 6 | 5 | 1 | 9 | 7 | 44 | 86 |
| Control | 1 | | 39 | | 40 | | 5 | |
| CP | 0 | 0 | 6 | 3 | 6 | 3 | 0 | 0 |

### ACKNOWLEDGMENTS

The authors thank Prof. Jivaldo Rosário Matos, for the thermo analysis, Fundação de Amparo à Pesquisa do Estado de São Paulo (FAPESP-94/1250-8) and the Conselho Nacional de Desenvolvimento Científico e Tecnológico (CNPq), for the financial support of this work.

### REFERENCES

1 - Rosenberg, B., *Cancer,* 1985, **55**, 2303.
2 - Bear, J.L., *Cancer Chemother. Rep.,* 1975, **59**, 611.
3- Zyngier, S.; Kimura, E. and Najjar. R., *Braz. J. Med. Biol. Res.,* 1989, **22,** 397-405.
4- Fimiani, V.; Aimis, T.; Cavallaro, A. and Piraino, P., *J. Chemother.,* 1990, **2**, 319-26.
5- Piraino, P.; Tresoldi, G. and Lo Schiavo, S., *Inorg. Chim. Acta,* 1993, **203**, 101-5.
6- Pruchnik, F. and Dús, D., *J. Inorg. Biochem.,* 1996, **61**, 55-61.
7- Souza, A.R., Najjar, R. and Glikmanas, S., *J. Inorg. Biochem.,*1996, **64**, 1-5.
8- Souza, A. R.; Najjar, R.; Oliveira, E. and Zyngier, S.B.I., *Metal-Based Drugs,* 1997, **4**, 39-41.
9- Joesten, M.D., Najjar, R., Hebrank, G. , *Polyhedron,* 1982, **1**, 637-9.
10- Goldstein, A., *Bio-statistics, an Introductory Text, 5.thed.,* Macmillan, 1967.
11- Deacon, G.B. and Huber, F., *Inorg. Chim. Acta,* 1985, **104**, 41.
12- Tajmir Riahi, H.A., *Carbohydr. Res.,* 1984, **125**, 241-8.
13- Parish, R.V., *NMR, NQR, EPR, and Mössbauer Spectroscopy in Inorganic Chemistry,* 1995, **2**, 27-92.
14- Horton D., Walaszek, Z., Ekiel, I. *Carbohydr. Res.,* 1992, **119**, 191-230.
15- Carper, W.R., Coffin, D.B., Addis, J.R., *Spectrochim. Acta,* 1989, **45A**, 391-2.
16- Coffin, D.B., Carper, W.R., *Mag. Reson. Chem.,* 1988, **26**, 591-4.
17- Escandar, G.M., Sala, C.F., *Can. J. Chem.,* 1992, **70**, 2053-7.
18- Heatley, F. , Scoott, J.E., Casu, B., *Carbohydr. Res.,* 1979, **72**, 13-23.

**Received: December 8, 1997 - Accepted: January 6, 1998 - Received in revised camera-ready format: January 4, 1999**

